# Novelty Knows No Boundaries: Why a Proper Investigation of Novelty Effects Within SHRI Should Begin by Addressing the Scientific Plurality of the Field

**DOI:** 10.3389/frobt.2022.741478

**Published:** 2022-05-27

**Authors:** Catharina V. Smedegaard

**Affiliations:** The Integrative Social Robotics (INSOR) Project, Department of Culture and Society, Aarhus University, Aarhus, Denmark

**Keywords:** novelty effects, social robotics, human-robot interaction, robophilosophy, interdisciplinarity, psychological novelty, sense-making

## Abstract

Research on psychological novelty effects within the fields of Social Robotics and Human-Robot Interaction (together: SHRI) so far has failed to gather the momentum it deserves. With the aid of exemplary descriptions of how psychological novelty is currently approached and researched across (certain main regions of) the larger scientific landscape, I argue that the treatment of novelty effects within the multidisciplinary SHRI reflects larger circumstances of fragmentation and heterogeneity in novelty research in general. I further propose that while the concept of novelty may currently function as a Boundary Object between the contributing domains of SHRI, a properly integrated, interdisciplinary concept of novelty is needed in order to capture and investigate the scope and scale of novelty effects within research on social human-robot interaction. Building on research on the New Ontological Category Hypothesis and related studies, I argue that the novelty of social robots can be understood as radical to the extent that their comprehension requires revisions of traditional core categories of being. In order to investigate the sui generis effects of such novelty, which should not be narrowly understood as mere “noise” in the data, it is paramount that the field of SHRI begin by working out a shared, integrative framework of psychological novelty and novelty effects.

## 1 Introduction

Reflections upon “novelty^1^” are not uncommon within the overlapping fields of Social Robotics and Human-Robot Interaction (henceforth taken together as SHRI, denoting research on Social Human-Robot Interactions). Within the last 10 years, several efforts have been made to gauge the impact of “novelty”—often also labelled the “novelty effect”—on the research data of SHRI (e.g., Leite et al., 2013; van den Berghe et al., 2019; Maj and Zarzycki 2019; Baxter et al., 2016). But have these scattered investigations been successful in addressing the full significance of novelty for SHRI? This is the question I wish to raise in this paper. My main purpose here is to reformulate the “question of novelty” for SHRI research by arguing for the following three claims: 1) Novelty research goes far beyond the domain of SHRI; 2) Treatments of novelty in other domains are highly relevant for SHRI; and 3) Aspects of novelty in the encounter with social robots should best be investigated with an understanding of novelty that is informed by such a wide-scope interdisciplinary perspective on the notion of novelty. Given space restrictions, I focus on presenting the positive message of what can be gained by operating with a more reflected notion of novelty that is informed by a wide-scope interdisciplinary perspective (what has been lost in extant research the reader can derive by implication).

I proceed as follows. In Section 2, I argue that currently, phenomena attributed to psychological novelty and novelty effects are approached and conceptualized within a wide variety of scientific fields surrounding SHRI, differing both in subject-material, level of analysis, focus, and methodological frames. When we hold these diverging novelty-expressions together, a fragmented and heterogenous landscape appears that falls short of conveying a deeper, more comprehensive understanding of the basic nature of the underlying phenomenon. In Section 3, I argue that social robots are radically novel, in that they force upon us radical disruptions of established sense-making. As such, the novelty effects at large within research on social robotics should be acknowledged as pervasive and central to the research target of SHRI. In Section 4, I try to make this suggestion more concrete by setting a few pointers for how and where SHRI research may benefit from greater attention to novelty and the cross-disciplinary variation and complexity of this notion. Most notably, I argue in Section 4.1 that the field of SHRI needs to overcome the larger circumstances of fragmentation and decentralization in research on novelty in general, and leave behind the predominantly narrow conceptions of novelty hitherto employed. I further propose in Section 4.2 that the most promising way to improve the current conditions for efforts on novelty research within SHRI is to begin by working out a shared conception of novelty and its behavioral and psychological effects that draws together and facilitates integration between the varying domain-specific perspectives of and approaches to the phenomenon.

## 2 Understanding Novelty Phenomena

One of the main aims of this paper is to argue in favor of a relatively open-ended enterprise of working out a more comprehensive, interdisciplinary understanding of novelty and novelty effects within SHRI. For this purpose, I need to make plausible 1) that such comprehensive approach is possible and 2) why it is recommendable. In this section I begin my argument by offering an overview of how different research domains outside of SHRI understand novelty, conceptually or operationally, displaying divergences and overlaps. As such, this section of the paper does not concern itself directly with SHRI research but serves as a necessary background for the SHRI-targeted arguments presented in the remaining sections of the paper.

The selection of accounts in the following overview is guided by three constitutive assumptions about psychological novelty phenomena that serve as heuristics for the entire argument:

1) Cause first, effects later: Whenever we investigate how novelty affects behavior and psychological states, our preliminary conception of novelty delineates what kind of effects we are looking for (and thus find). Therefore, in order to understand the possible variety and significance of novelty effects, we need to begin by understanding the basic characteristics of the novelty-kind we are concerned with.
2) Focus on psychological novelty: When we concern ourselves with the kind of novelty that by being novel can affect what people think and do, we are concerned with psychological phenomena. This means that while something may be argued as ontologically, objectively, historically, scientifically, etc.^2^, novel, this status will be largely irrelevant if the responding person(s) do not also perceive it as such. As far as novelty can affect any kind of behavior or psychological state, it needs to be identified as such through a mental process (Berlyne and Parham 1968; Habib 2000; Witt 2009; Förster et al., 2010). Therefore, a comprehensive conception of psychological novelty and novelty effects needs to set out with an understanding of novelty as a psychologically-realized property.
3) Focus on experience and sense-making: (Human) psychological novelty as an “effect-instigator” involves an experiential dimension; it involves the experience of lacking familiarity with something^3^. Vast amounts of trivial novelty surround us every day, in the form of cups we have never drunk from before, chairs we have never sat in before, or mails we have never written before. But it is only when something somehow disrupts our experience of automaticity or familiarity, or invokes a sense of deviance, that our thoughts and behaviors are affected and the experience of novelty is realized. In the pursuit of understanding psychological novelty effects, it is important to explore the role of this experiential dimension, lest we lose sight of the kind of novelty that has high practical significance. We need to try to understand the characteristics of novelty in relation to the experience of our ongoing activities of sense-making, and allow for broader understandings of concepts such as knowledge, memory, familiarity, learning, etc., than is possible within the terminologies of biology or cognitive science.

As I have argued elsewhere (Smedegaard 2019), with suitable supplementations these three assumptions point towards a new research target for psychological novelty research in general—I have called it “experiential novelty”. For now, however, they serve as the heuristic framework within which the following traditions of novelty research have been identified and juxtaposed. I will henceforth refer to psychological novelty as simply “novelty”, taking the specification of “psychological” as implicit unless otherwise specified.

### 2.1 Diverging Constructs of Novelty in Science

There is a plethora of rich and lively traditions of novelty research, and any selection does injustice to the complexity of criss-crossing thematic connections, within and across disciplines and domains^4^. In the following I have collected treatments of novelty from within domains that all, in some fashion or other, center on, or rely on, psychological phenomena—we may call them “psychology-reliant” domains of research^5^. The themes that I highlight in such “psychology-reliant” approaches to novelty are nothing else but that; themes and contrasts, not critical and exhaustive evaluations. While the respective treatments of different research tracks in novelty research are well-motivated in their own right, when highlighted in cross-disciplinary perspective, contrasts appear once implicit contextualizations and practical embeddings are not accessible. The contrastive synopsis I offer here is meant to illustrate both 1) the vast knowledge-resources and perspectives on novelty available if one is willing to look beyond one’s own immediate scientific embedding, and 2) the tensions and idiosyncrasies that need to be addressed, if a more comprehensive or more integrated understanding of novelty is to be achieved.

#### 2.1.1 Classical Psychology: From Surprise to Subjective Novelty

In classical psychology, the idea of novelty as something that affects behavior can be traced back as far as to the origins of conditioning theory and the observation of “the Orienting Response” (OR) (Pavlov, 2010). In this early conception, novelty was largely used synonymously with surprise, emphasized as something that disrupts and “grabs” attention. However, between the 1950s and 1970s, experimental psychologist Daniel E. Berlyne advocated repeatedly, as one of the first, for more precise definitions and measurements of “subjective” novelty as a psychological phenomenon. He argued, inter alia, that the concept of novelty had so far been confused with “other properties, such as change, surprisingness, and incongruity” (Berlyne and Parham 1968, p. 415), and that a main challenge in defining (human experiences of) novelty was the complexity with which it could arise and exist in a number of different forms or types (Berlyne 1950; Berlyne 1960).

#### 2.1.2 Neurocognitive Science: Back to Surprise But Diversified

Nevertheless, empirical operationalizations of novelty as instances of surprise have endured the orientational shift from behaviorism onto the information processing paradigm in the latter half of the twentieth century (Sokolov 1990), for instance with research on Event-Related Potentials (ERPs) positing the “Novelty P3” (explained very simply as a cluster of spikes in electrical brain-activity about less than half a second after the onset of a stimulus) as the neurophysiological equivalent to the Orienting Response (Friedman et al., 2001; Barry et al., 2016). In general, within research on both non-human and human cognition, the phenomenon of novelty has now been vastly investigated in relation to subjects such as perception, attention, memory, learning, and decision-making, emphasizing the fundamental role of novelty in how cognizant organisms discriminate and process the incoming stream of information from its surroundings. The majority of accumulated neurocognitive research on novelty assessment and processing focuses on testing-paradigms that employ instances of novelty based either on being “atypical” or “deviant” from the rest of the presented stimuli, or on not having been presented before within the context of the study (Habib 2000; Barto et al., 2013). Furthermore, due to the level of investigatory focus, the stimuli presented are traditionally in the form of simple, nonsensical words, syllables, pictures, sounds, etc., that usually do not require more extensive repetition or engagement in order to become familiarized (e.g., Knight 1996; Stern et al., 1996; Kormi-Nouri et al., 2005). Thus, the “immediate” or “instant” response to or recognition of novelty becomes the “entry-point” in a way that makes understandable why descriptions of surprise, disruption, and attention-grabbing remain the preferred definitions of novelty in this research track.

A lack of consistent definitions of novelty, hereunder the problematic conflation of novelty with surprise, has been re-iterated in more recent times by several researchers within neurocognitive science (Habib 2000; Kagan 2009; Barto et al., 2013). Neuroscience research operates with several novelty-kinds which are, however, not always clearly distinct nor used unequivocally: for example, taking Bünzeck and Düzel (2006), Kagan (2009), and Barto et al. (2013) combined, we are offered definitions for six different kinds of novelty, i.e., “stimulus novelty,” “conceptual novelty,” “associative novelty,” “contextual novelty,” “absolute novelty,” and “relative novelty.” However, when comparing their uses across the three articles, none of them are consistently or unanimously defined. The challenge of how to define and measure novelty within neurocognitive research poses equal challenges to the enterprise of mapping the brain areas and functions responsible for novelty detection and assessment. Presumably as a result of divergent notions and operationalizations of novelty (and its relation to its opposite, familiarity) there currently exists divergent theories and data sets on the processes underlying novelty detection (Habib 2000; Habib et al., 2003; Barto et al., 2013) and novelty reduction, such as priming, repetition, and habituation (Habib 2001; Rankin et al., 2009; Konkel 2012).

#### 2.2.3 Comparative Psychology: Surviving Through Exploration and Avoidance

Within comparative psychology, the detection of and response towards novelty is largely viewed from a perspective of survival and adaptation (e.g., Salomons et al., 2010; Kozlovsky et al., 2015). Here, novelty occurrences have historically been equaled instrumentally with instances of threat or reward, narrowly triggering responses of either avoidance or exploration (e.g., Blanchard et al., 1974; Molas et al., 2017). In this field too, operationalizations of novelty cover both unexpectedness, disruption and unfamiliarity; in studies of novelty as unexpectedness and disruption, focus is mainly on immediate responses of exploration or avoidance, whereas cases of novelty as unfamiliarity seem to focus to a larger degree on the resulting longer-term familiarization and forming of novel behaviours (e.g., Kaufman et al., 2011). The terms “high and low-responders” are used to denote levels in sensitivity towards novelty, and historically, as in many other cases within comparative psychology, the rat in particular has been a preferred test subject, with a special, highly novelty-sensitive type of rat, the Nijmegen breed (Saigusa et al., 1999), having been bred in order to observe more clearly how neurochemical profiles and behaviours change in relation to novel stimuli. Predominantly, the emotional valence of novel stimuli (as either desirable or aversive) has been understood as dependent upon innate, stable traits, in terms of neophobia or neophilia, from the assumption that there are species-specific predispositions to react to novelty with either aversion or approach (Wood-Gush and Vestergaard, 1991; Greggor et al., 2016). Considering the obvious challenges in gaining direct access to the “experiential” or “subjective” realm of animal psychology, research on the contents of animal cognition has been predominantly indirect, based largely on neurophysiological and behavioristic paradigms (Réale et al., 2007). However, more recently, some have voiced concern with this underdevelopment of investigatory focus, arguing that it has led to heterogenous measurements and incomparable data within research on animal responses to novelty (Greggor et al., 2015). They further argue the need for defining and studying expressions of neophilia and neophobia from a more integrative and nuanced paradigm that emphasizes the role of both context, type of novel stimuli, and cognitive “configurations” of the animal, in forming a given response to novelty (Greggor et al., 2016).

#### 2.2.4 Motivation Research: Novelty as an Innate Need

In research on (human) motivation, novelty is framed as something intrinsically desirable, e.g., evaluated not by its instrumental role in honing in on rewards or threats, but as an end-goal in itself. In their Self-Determination Theory (SDT), Ryan and Deci (2000); Deci and Ryan 2000 describe intrinsic motivation as an “inherent tendency to seek out novelty and challenges, to extend and exercise one's capacities, to explore, and to learn” (Ryan and Deci, 2000: 70), and identify three basic, innate needs for competence, autonomy, and relatedness acting as regulators of this tendency (Deci and Ryan, 2000); when satisfied, intrinsic motivation-levels are high, and when not, this kind of motivation is subdued. Implied by this theory is novelty as something originally desirable, that only in times of strain and forestallment of needs becomes aversive. This implication has been made explicit by the later suggestion that novelty should be recognized as a fourth, innate need (González-Cutre et al., 2016). Again here, “novelty” denotes occurrences of both unfamiliarity and occurrences of deviancy alike [as something that “deviates from everyday routine” (Ibid.)].

#### 2.2.5 Personality Research: Novelty as an Individual Preference

In contrast to this stands research on personality traits, where the emotional valence of and reactions towards novelty are investigated not as intrinsically determined, but as dependent upon the temperamental disposition of the individual. For instance, in Costa and McCrae’s Five Factor Model (FFM) of personality (Costa and McCrae, 1992a; Costa and McCrae, 1992b), the traits of Openness to Experience and Extraversion are often specified in combination as reflecting the degree to which an individual positively engages with and seeks out novel experiences (e.g., Gordon and Luo, 2011; Goclowska et al., 2019). Similarly, in Cloninger’s Psychobiological Model of Temperament and Personality (PMTP) (Cloninger, 1994), the temperamental dimension of Novelty Seeking denotes the frequency with which an individual engages in exploratory behaviors and approaches novelty. Measures of personality-based responses to novelty often include measures of tendencies towards risk-taking, sensation-seeking, and reward-dependence, leading to the frequent equation of novelty with situations marked by heightened danger or harm (Goclowska et al., 2019), such as drug use (Wingo et al., 2016) and extreme sports (Myrseth et al., 2012); for instance, while Cloninger operates with a dimension of harm avoidance, thus separating the tendency of seeking novel experiences from the tendency to be outgoing and risk-taking [defined as low harm avoidance (Cloninger, 1994)], comparisons between Cloninger’s PMTP and Zuckerman’s Sensation-Seeking Scale (SSS) reveal correlations between the Cloninger’s Novelty Seeking and Zuckerman’s Sensation-Seeking, raising doubt as to whether Cloninger’s operationalization of Novelty Seeking actually denotes a larger affinity with seeking risks and high arousal than with unfamiliarity per se (McCourt et al., 1993; Zuckerman and Cloninger, 1996). Similarly, in the FFM, novelty attitudes are usually measured through a combination of Extraversion and Openness to Experience. Yet, the fact that these dimensions also designate attitudes toward other circumstances (for instance, Excitement Seeking) makes it hard to isolate the distinct characteristics of novelty in itself as a target of individual preference (Aluja et al., 2003).

#### 2.2.6 Curiosity Research: Novelty as Potential Expansion of Knowledge

Closely related to both motivation and personality research, is the subject of curiosity. Both early (Berlyne, 1960) and more contemporary research (Kashdan et al., 2009; Kashdan and Silvia, 2009) on curiosity denote the ambiguity of novelty as both an anxiety-inducing and interest-eliciting experience; while novelty [framed as the possibility to learn or experience something unknown (e.g., Van Dijk and Zeelenberg, 2007)] has often been posited as the object of desire for curiosity through the conflation of curiosity with both novelty-seeking or intrinsic motivation (Kashdan et al., 2009), Berlyne (1960) and Kashdan et al. (2009) stress that curiosity does not simply involve the desire to experience novelty, but also (and quite importantly) depends upon individual thresholds of tolerance towards uncertainty. In this way, the degree of uncertainty accompanying a novel experience is made pivotal to the emotional appraisal of it (Loewenstein 1994).

#### 2.2.7 Innovation, Consumer, and Marketing Research: Novelty as Both Product and Value-Production

In another branch of psychology-reliant research, the overlapping areas of innovation, consumer, and marketing research are also heavily concerned with the phenomenon of novelty. Here, novelty is viewed as central to both product development (Lewis and Bergin, 2016), product value (e.g., Dewett and Williams, 2007), and consumer behaviors (e.g., Hirschman, 1980), predominantly as something extremely desirable. In Diffusion of Innovations from 1962, Rogers (1983) presented his now extensively adopted theory on how “innovations,” defined as “an idea, practice, or object that is perceived as new by an individual or other unit of adoption” (Rogers, 1983: 11), are spread throughout social systems. Currently, the term “innovation” is used broadly to specify a wide range of products or processes deemed to be both new and valuable or beneficial within a given context (e.g., Coopey et al., 1997). However, some have argued that there has been a tendency to emphasize the importance of novelty whilst overlooking the functional aspects of value or benefit, thus resulting in innovation-approaches too focused on developing and supporting novelty without regard for the durability of the enterprise (Janssen et al., 2015; Tanggaard and Wegener, 2016). Similarly, research on creativity posits the role of novelty as both an “instigator” of creative behaviours (Kaufman et al., 2011; Gillebaart et al., 2013), and as an inherent property of creative outcomes (Dean et al., 2006; Guegan et al., 2017), with researchers likewise noting that while the predicate of being “creative” is often equaled to being novel, creativity may further involve an aspect of usefulness or value that novelty in itself does not necessarily procure (Dean et al., 2006; Kaplan and Vakili, 2015). Research on innovative products, often in relation to technology, discriminate between instances of incremental and radical innovation; the former denotes smaller-scale improvements of and add-ons to already existing functions and products, while the latter denotes inventions and advancements that break with established traditions of practice and require the formation of novel skill and knowledge (Dahlin and Behrens, 2005; Norman and Verganti, 2014).

Within consumer and marketing research, the notion of novelty as intrinsically desirable is effectively upheld both by the identification of “novelty-intensive markets” where demand for a product is based mainly on its novelty (Dewett and Williams, 2007), and by the existence of large numbers of studies on how to enhance consumer experiences of product novelty, for instance through kinetic properties in visual advertisement (Kim and Lakshmanan, 2015), or through ways of framing new products in relation to existing products (Ziamou and Ratneshwar, 2003). In general, measures of novelty responses within consumer research count numerous different approaches to assessing aspects of consumers’ readiness to adopt novel products (e.g., Hirschman, 1980; Hoeffler, 2003). In addition, investigations of more market-specific novelty responses are in themselves a study the kaleidoscopic variety of novelty phenomena, owing to the fact that responses are investigated in relation to practically anything that is marketable; for instance, within the food sector, a highly specific response to novelty known as food neophobia is of concern (Barrena and Sanchéz, 2012; Faccio and Guiotto Nai Fovino, 2019); within tourism, the role of novelty in the formation of “memorable tourism experiences” is sought clarified (Skavronskaya et al., 2020); and in the context of theme parks, the degree of perceived novelty of the parks’ physical surroundings is investigated in relation to customer behaviors of buying and re-visiting (Chang et al., 2013).

#### 2.2.8 Liminality Research; Novelty as Possibility

Lastly, at a much more experience-oriented level, research on the anthropology-derived concept of liminality deserves mentioning. Originally inspired by the progression of relatively universal transitions—or “rituals of passage”—throughout common life (Van Gennep 1960), the concept has in modern times been employed to denote more broadly the transactional state that arises from moving or being in-between the borders of different positions or situations of meaning (Ybema et al., 2011; Teodorescu and Cálin 2015; Picione and Valsiner 2017). Research on this concept employs a view of novelty and change as something “undetermined” or “yet-to-be-determined”; as an individual transitions from one set of established circumstances of meaning into another, she finds herself in a liminal space of ambiguity and uncertainty, where the “bonds of what was”’ are untied and new bonds form. The existence of liminal spaces is not limited to the progression from the known to the unknown, but are equally found in transitions between different known positions. However, while the movement between two known positions can be understood as a process of transformation, the movement from the known to the unknown also involves an element of original formation as the novel circumstances themselves—that which comes after—are formed by and emerge from the processes of (re-)construction and (re-)interpretation occurring in this liminal space (Beech, 2011; Picione and Valsiner, 2017). Within the frames of liminal processes of meaning-making, novelty is thus emphasized as circumstances of potentiality and possibility.

### 2.2 Novelty Constructs as Heterogeneous Expressions of the Same Phenomenon?

The preceding synopsis illustrates how novelty relates to core aspects of human nature. Nonetheless, many areas of research with explicit notions of novelty have been left out: for instance (and not surprisingly), novelty is widely researched in relation to the subject of learning, both within and outside educational domains and on many levels of analysis; within machine learning, the algorithmic definition of novelty figures as crucial to open-ended learning, the generation of novel output, and the detection of anomalies (Lehman and Stanley, 2011), inter alia; within clinical psychology, responses to novelty are studied in relation to mental disorders, such as ADHD and schizophrenia (Molas et al., 2017); and within organizational theory, research on organizational sense-making involves notions of novelty as events of disrupted sense-making (Weick et al., 2005). In addition, with novelty being equaled broadly to circumstances of change, surprise, disruption, incongruence, atypicality, deviance, creativity, innovation, uncertainty, unfamiliarity, etc., it stands to reason that many more psychology-reliant research subjects and areas may involve, albeit perhaps more implicitly, aspects of psychological novelty.

Despite its limitations, I trust the preceding synopsis can convey that psychological novelty permeates a wide range of research areas devoted to investigating on how human beings come to know and engage with their surroundings. But can we distill a unified notion of “novelty” from the plethora of research targets and domains investigated? Considering both the ease with which we normally intuit what is meant by the term “novel” (to the point where even many academic treatments omit an actual definition), and the recognizable, recurring convergences between the many novelty-treatments, it seems reasonable to assume that all these different notions of novelty share a common denominator. If so, then these notions can be understood as subject-, domain-, or discipline-specific expressions or tokens of an underlying core phenomenon or type. However, despite apparent commonalities, these novelty-expressions are also diverse constructs of larger systems, placed at different levels of analysis and attached with different terminologies, measurements, theoretical trajectories, and observational foci^6^. Thus, if we take for granted that we largely mean the same thing by the term “novelty” across disciplinary and topical borders, attempts to translate and abstract across these borders risk turning originally context-based choices of demarcation into blind angles or confusions (perhaps even misconceptions) about the nature and role of novelty in broader contexts—as has similarly been the case for the concept of the “gene” within subfields of biology (Flodin, 2009).

To the best of my knowledge, there has yet to be developed a broader, conceptual model of psychological novelty that explicates and defines its nature (and hence effects) as an underlying source of all the different novelty constructs throughout the landscape of (psychology-reliant) novelty research. This is not to say that there have not been efforts to draw out and specify the elusive nature of novelty at more foundational and conceptual levels (see for instance Witt, 2009), but of the proposals I am aware of, they are all themselves delimited by field- and subject specifics, and as such do not ascend to a more general metadisciplinary perspective. Without the guidance and translation from a unifying framework from which to identify, map out, and relate the similarities and differences between these diverse constructs, scattered and at-level comparisons between particular instances of approaches, measurements, definitions, etc. may not necessarily yield deeper comprehension of the actual complexity and extent of psychological novelty and novelty effects. That the need for such a framework has not been voiced yet can be taken to suggest that the existing supply of novelty constructs are deemed as sufficient for the respective disciplinary research enterprises. However, in the next two sections of this paper, I return attention to the field of SHRI and argue that research on social robotics needs to address novelty-phenomena of such scope and scale that it requires the development of a larger, interdisciplinarily shared conceptualization of novelty in order to accommodate this need and comprehensively investigate and understand these phenomena.

## 3 The Novelty of Social Robots: The Significance of Novelty Within SHRI

The survey offered in the preceding section conveys the definitional heterogeneity of the notion of novelty, but also that a good number of approaches to novelty intersect in linking novelty to disruption, unfamiliarity, knowledge-generation—i.e., processes that in some fashion or other relate to a disruption of sense-making. Taking this as a sign that this is a central aspect of novelty, I will show in this section that social robots—while perhaps failing to be novel in some other senses of the term—present us with a profound and radical kind of novelty if we understand the latter as the disruption of sense-making. I begin by a short elaboration of this interpretation of novelty.

### 3.1 Psychological Novelty as Disruption of Sense-Making

“Disrupted sense-making” is the label for a complex of cognitive processes which we can bring into view again by selecting three characterizations of novelty that operate at different levels of analysis and yet describe what appears to be one phenomenon from different perspectives, each supplementing further details.

1) Within classical information-processing interpretations of neurocognitive data, novelty-detection and assessment theories basically assume that novelty is detected when the comparison of incoming stimuli fails to find matching contents within long-term memory storage (Tulving and Kroll, 1995; Barto et al., 2013). This detection sets off a collection of behavioral responses (that can largely be divided into either exploration or avoidance) as well as cognitive memory-formation processes, that decrease in activity as the stimuli is repeated and familiarized (Tulving et al., 1996; Ranganath and Rainer, 2003; Kormi-Nouri et al., 2005).
2) Within the alternative, fast-growing area of action-based approaches to cognition, most notably through the accounts of Situated Action (Barsalou et al., 2007) and Embodied Cognition (Bar, 2007; Cisek and Kalaska, 2010), it is generally theorized that (much of) human cognition is aimed at providing the subject with a real-time foundation for action, meaning that “knowledge” is continuously activated and generated on basis of a need to adapt to and act efficaciously within one’s environment. In situations where existing knowledge fails to provide efficient (Bar, 2007), correct (Barsalou et al., 2007), or consistent (Proulx et al., 2012) inferences to act upon, behaviours and cognitive processes are set in motion that ultimately generate additional knowledge from which to draw new inferences from (Barsalou, 2008).
3) Within experientially-focused theories on narrative-building within semiotics and psychology, it is suggested that humans understand their continual interactions with their environment through the constant formation of contingent narratives that serve to bind the moments of their lived lives together into a meaningful whole (Brockmeier, 2009; Picione and Valsiner, 2017). When routine or habitual narratives are disrupted, in the sense of no longer providing a meaningful whole, efforts are made to restore meaning by adjusting and creating alternative narratives that can mend the broken flux.
4) In addition to the three procedural characterizations, this fourth characterization denotes scale through the distinction between “incremental” and “radical” novelty, previously presented in Section 2. In literature on technological, scientific, and organizational invention and change, “incremental” describes products or developments that build upon and “fine-tune” already existing structures and trajectories of praxis, tradition and meaning (McAdam, 2003; Dahlin and Behrens, 2005; Norman and Verganti, 2014). However, some advancements or changes involve circumstances that are so far beyond the status quo that their integration requires a revision of, or even a break with, certain traditions of thought and/or praxis (McAdam, 2003). In a Kuhn-inspired interpretation, these resultant changes are radical in the sense of paradigm-shifts, as they disrupt established meaning within a given context and open up hitherto inexistent or unexplored domains of action and sense-making (Rogers, 1983; Kuhn and Hacking, 2012). In this interpretation, some disruptions of sense-making may only require smaller adjustments or add-ons to an existing paradigm or narrative of meaning in order to become integrated, while others may require the emergence of entirely different paradigms or narratives in order to be comprehended and made sense of. As such, radicality pertains to the kind and depth of the knowledge-disruption that an experience of novelty is realized by, and not to the intensity of the affective response to this disruption (which should be considered a novelty effect)*.*

Together, these four characterizations describe a link between novelty and knowledge (in its widest sense, from perceptions of simple stimuli, through conceptions of actionable environment-features, onto the continual experience of one’s situation as a meaningful whole) that effectively frame the cognitive and behavioral consequences of encountering novelty as processes of knowledge-generation—or, in a broader frame of experiential novelty, processes of sense-making—at times when already established knowledge is found inadequate.

### 3.2 Why Social Robots Are Radically Novel

By a “social robot” I understand a technological social agent that through embodied presence and social affordances gives rise to the perception, at least temporarily, of “being-with” a social partner rather than of employing a functional tool (Seibt et al., 2020) Already at our current stage of “automation,” the experience of novelty that an encounter with a social robot elicits in people with normal cognitive functions is, as I will try to show now, best understood as a disruption of sense-making.

More precisely, I argue that social robots are radically novel in that they force upon us radical disruptions of established sense-making (henceforth, shortened to “disrupted sense-making”), since the experience of social robots as novel lies in their experienced combination of attributes that effectively cut across foundational core conceptual distinctions of animate vs. inanimate or living vs. non-living categories of being^7^. Regardless of approach to cognitive organization of representations, general consensus posits that human conceptual knowledge seems to operate with some “core” categorical distinctions between the kinds of phenomena we encounter in the world (Barsalou, 2003; Cisek and Kalaska, 2010)^8^. The categories of “animate and inanimate” and “living and non-living” are usually understood as such core distinctions (Mandler, 1992; Jolly, 2011; Kahn and Shen, 2017), and it seems further plausible that the distinction between conscious vs. non-conscious should lie at the same level. Experienced breaks with these common distinctions, including the conscious/non-conscious, have been observed by a good number of studies in SHRI research (e.g., Severson and Carlson, 2010; Welge and Hazzenzahl, 2016; Kahn and Shen, 2017; Damholdt et al., 2019; de Graaf and Malle, 2019), who all found that social robots seem to be classified, by the same individuals, explicitly or by implication, as simultaneously “animate/living” and “inanimate/non-living”—or “alive enough” (Turkle, 2011: chap. 2)^9^. Henceforth, I will refer to these categories broadly as “living” and “non-living”. As Kahn et al. (2011) effectively sum up, in relation to their proposal of the New Ontological Category Hypothesis;

“In brief, there is emerging evidence to suggest that there is a constellation of attributes that children and adults ascribe to personified robots—including those that involve mental states, sociality, and in some ways even moral regard—which do not appear to mirror reasoning about such canonical living entities as humans, non-human animals, or artifacts” (Kahn et al., 2011: 160).

It thus appears that social robots currently give rise to experiences of co-existence between properties of both living and non-living in a way that breaks with foundational assumptions of them as mutually exclusive. Moreover, because these properties are usually ingrained as the deciding features of said categories, it is plausible to assume that they elicit a state of uncertainty that cannot be remedied solely by means of adding on to what is already known. Instead, it seems that the conceptual comprehension of these new agents requires both the invocation of a third representational category of beings somehow “alive and not alive at the same time,” as well as a suspension of the existing category-properties’ exclusivity—or in the sense of radical novelty above, a change in paradigm or narrative.

Perhaps this change is only temporary: the inherent uncertainty of psychological novelty—that we simply do not know yet what we are experiencing—also means that we do not know in advance how to best make sense of it (Witt, 2009). In this sense, we are in a liminal space of potentiality where future interpretations have yet to be decided. It is fairly well-established that in the face of uncertainty people are capable of entertaining several narratives or lines of reasoning at once. For instance, the Novelty Categorization Theory (Förster et al., 2010^10^) proposes that people engage in global processing strategies when experiencing novelty, in order to broaden the scope of cognitive categories and increase the possible associations between them; when novelty has become familiarized and integrated, cognition returns to local processing. Similarly, it has been proposed that as people are capable of going back and forth between paradigms of meaning, Ibrahim A. Halloun’s notion of mental paradigms as flexible and “convertible” is a more fitting analogy for human reasoning than Kuhn’s original, one-way paradigms (Wendel, 2008). As such, the radicality of robot novelty lies not in whether they will in fact become accepted as a third category of being, like Kahn and colleagues propose (Kahn et al., 2011; Kahn and Shen, 2017), but in the fact that we feel the need to entertain the thought—and by it, question fundamental assumptions about what it means to be living; to be conscious; to be social; to be a caregiver; to have rights; to have responsibilities; etc. In another striking passage, Kahn and Shen (2017) write:

“Granted, for a while more, we might continue to ask such questions as, “Are these robots fundamentally alive or not alive?” or “Do you see these robots as more social or more technological?” It’s like we hardly know how to ask the right questions, because we (as adults) are stuck trying to get our minds around a new ontological form using the “old” ontological categories we ourselves constructed as children” (Kahn and Shen 2017: 119).

The significance of the novelty of social robots lies not in whether people will continue to experience robots as in between, but instead in the fact that they do so now. In the above quote, Kahn and Shen effectively describe the conditions of psychological novelty as disrupted sense-making, although to them it is merely a waiting-position while the ontological status of social robots emerges.

In sum, then, social robots present a radical case of novelty as disrupted sense-making for two reasons. First, the meaning that is disrupted involves binary classificatory notions which provide basic orientations in our sense-making, such as “alive—not alive” or “conscious—unconscious.” Second, the disruption creates deep cognitive uncertainty that is actively responded to by continued efforts at sense-making. (It is this second component of the novelty of robots that might partly explain why we continue to find robots so curiously intriguing, as documented in the long history of automation).

I will in the rest of this paper refer to this particular account of the novelty of social robots as radically disrupted sense-making as the RDSM-approach. I now turn to explicate four beneficial implications of working with this approach within SHRI.

## 4 Four Reasons for Increasing Interdisciplinary Efforts on Novelty Research Within SHRI

If social robots can be considered to engage us in an experience of radical psychological novelty, understood as a profound disruption of sense-making, what are the implications? Here I wish to set out four reasons for why it can be beneficial for SHRI to treat novelty as an important dimension of human-robot interactions.

### 4.1 First Reason: Aiding Comprehension of the Pervasiveness of Novelty Effects Within SHRI

The phenomenon of novelty effects is by no means an ignored concern within the SHRI community. Yet, in early SHRI-literature, the issue has been mostly addressed as a peripheral, brief, and often post-study reflection upon the degree to which findings may have been affected by novelty effects, either not further specified (e.g., Michaelis and Mutlu, 2018; Cifuentes et al., 2020) or as simply increased levels of affective arousal or interest (e.g., Gockley et al., 2005; Breazeal et al., 2016)—the advice from Kidd and Breazeal (2005) to simply let participants interact a little with the robot prior to data collection “to reduce novelty effects if that is a strong concern” (Kidd and Breazeal, 2005, pp. 142), reflects this dominant conception of novelty effects as noise in need of reduction effectively (for a further account of this “noise”-conception within SHRI, see Smedegaard, 2019). Since then, more distinct efforts to address novelty effects have surfaced, such as (Leite et al., 2009; Leite et al., 2013; van den Berghe et al., 2019; Maj and Zarzycki, 2019; Baxter et al., 2016) where (mechanisms underlying) novelty effects are acknowledged and/or treated as an important phenomenon worthy of closer investigations. Still, in comparison to other related “hot” topics in SHRI research such as anthropomorphism (Epley, 2018), long-term engagement (Leite et al., 2013) and the Uncanny Valley (Cheetham, 2017), novelty effects have so far failed to gather momentum as a dimension of social human-robot interaction on the shared research agenda in its own right. For instance, to my knowledge, so far there are no survey or review-articles with the explicit main aim of identifying and classifying common characteristics of psychological novelty phenomena across the SHRI publication-history. No workshops, symposia, or conferences on the topic have been held, and the present article collection is the first to address novelty effects as a special issue.

Together with a broadened comprehension of the multitude of ways in which novelty can be approached (offered in Section 2), novelty-conceptions such as the RDSM-approach help to remedy this situation by expanding and elucidating the role of novelty within SHRI. With the notion of social robots as instances of disrupted sense-making, it is possible to glean the equal radicality of the novelty effects currently taking place. While the signs of this disrupted sense-making may not be immediately observable (e.g., in the sense of being simply extreme exploration or avoidance), the effects, understood as processes of generating new meaning, should still be considered radical as they involve the entertainment of narratives or paradigms significantly different from what was pre-disruption.

Because much (if not all) of our current research within SHRI is basically aimed at understanding how social robots can and should be made sense of now and in the future, valuable sources of knowledge will continue to be foregone if investigations into novelty effects remain isolated, scattered and without scope. As de Graaf and Allouch (2016) nicely put it:

“Some scientists foresee that the long-term use of robots will change society in different aspects […]. We need to attend to these issues rather sooner than later if we want to anticipate on the (negative) consequences. As technology evolves, it impacts society regarding the beliefs, expectations and attitudes of people […]. By the time the societal impact of the rise of robots can actually be assessed, robots already have been integrated into our society.” (de Graaf and Allouch 2016: 755)

Understanding novelty as instances of disrupted sense-making, it becomes important that we investigate how people currently appraise and mend the broken flux in their engagement with these robots. Some of the resulting attitudes, reactions, interpretations, and narratives may develop into stable, or even permanent, responses, while others may be abandoned or change as experience with the robots increases. As such, quantitative attention to behavioral output must be supplemented with qualitative attention to the cognitive and experiential processes underlying this output (e.g., De Graaf et al., 2017; Damholdt et al., 2019). Because we ultimately cannot know yet which responses will last, gaining a deeper understanding of these novelty responses provide us with unique opportunity to both investigate in-depth the precursors of more permanent responses, as well as to aid as best we can those responses, we believe most responsible and sustainable in the future. de Graaf and Allouch (2016) argue that we need to include people’s perceptions of future robotic usages in current robot design and research processes in order to secure a positive future integration within society. In addition, I argue, by investigating and understanding the processes of sense-making currently in play when people engage with (even just the notion of) social robots, we can utilize this knowledge in forming already now the responses that we wish to enhance in the future.

For instance, research in cognitive dissonance finds that once people commit to a certain action-choice, they also engage in consolidating cognitive efforts to justify this choice, sometimes resulting in attitude changes (Harmon-Jones et al., 2015). This suggests that when a given research situation (intentionally or not) “pushes” participants to interact in a certain way with a robot, this may also implicitly “push” their attitudes and reasoning in certain directions, thus shaping how they think and feel about the robot. Conversely, as I have just described earlier, people are also able to entertain several differing lines of reasoning at once, meaning that in other cases, participants may be operating with several interpretations of the robot without necessarily feeling the need to “commit” definitely to any of them (Severson and Carlson, 2010). What determines whether an individual will respond to novelty with the need to resolve a cognitive dissonance quickly or to explore further before deciding, is precisely the kind of knowledge that needs to be utilized in SHRI research on novelty effects. Continuing the example, I have previously theorized that both the intensity and complexity of the novel occurrence seems to affect the emotional appraisal of it (Smedegaard, 2019), thus affecting the degree to which people choose to engage curiously and flexibly (exploratorily) with the experience (see also “curiosity research” in Section 2). If so, then we need to further investigate what determines the subjectively experienced intensity and complexity of engaging with a social robot. At least, it seems plausible to assume that being asked to assess potential interactions scenarios with a robot based on a picture alone elicits a state of uncertainty much different than an actual case of negotiating a physical space with a robot. Understanding what decides whether people respond curiously or apprehensively, naturally or with great effort, may also help to explain differences in participant responses across studies (further discussed in Section 4.3)

Therefore, if we are successful in leaving behind the predominant assumption that novelty effects are mainly just peripheral or short-term noise (Smedegaard, 2019) and instead commit to a much broader conception of novelty effects as a rich category of psychological and behavioral phenomena relevant to how human beings work their way out of uncertainty, I believe that we will find the role of novelty to be much more extensive and utilizable within the activities of SHRI than currently realized.

### 4.2 Second Reason: Novelty Research Can Serve to Foster the Interdisciplinary Integration of SHRI

While it has been clear from early on (Dautenhahn, 2007) that no single, already existing discipline or domain of knowledge by itself will be able to provide the total set of concepts, theories, and tools needed in order to fully grasp, or make sense of, the phenomena and consequences generated by robot sociality, there is an ongoing methodological debate as to whether the field of SHRI needs to transform from a multidiscipline to an interdiscipline or even transdiscipline, in response to the rising demand for sharing and synthesizing knowledge and methodologies across disciplinary and domain boundaries (Hillan, 2005; Breazeal et al., 2016; Baxter et al., 2016; Damholdt et al., 2019). The radical novelty of social robots provides another pressure for this transformation: since the experiences generated by social robots cut across experientially solidified^11^ core-distinctions in human knowledge organization, SHRI must integrate the expertise needed to model these cross-cuts theoretically, and explicitly engage them in empirical research. In the words of the previous section, social robotics opens up a hitherto inexistent domain of phenomena that bears upon core subjects within already existing knowledge domains across the scientific landscape in a way that requires reinterpretations and revisions of (some of) the contents of these domains. Although the differences between various formats of pluri-disciplinarity are not unanimously agreed upon, one way to roughly distinguish between them is through differences in levels and outcomes of knowledge-integration (Youngblood, 2007; Nersessian and Newstetter, 2014; Nicolescu, 2014): roughly, multidisciplinarity marks scientific collaborations where researchers come together in solving a specific problem, employing and combining relevant methods and theories from each area, and then returning to their respective areas which remain largely unchanged by the temporary collaboration. Interdisciplinarity marks collaborations that, on basis of synthesis between the collaborating areas, give rise to the development of new, hybrid theories and methods, and perhaps even a new hybrid area of research altogether. However, the original areas still remain largely unaltered. Lastly, transdisciplinarity marks collaborations that, through synthesis and integration of theories and methods, give rise to new knowledge, practice, and research areas in such a way that parts of the original areas are equally changed with lasting effect. In this description, it makes sense to view the potential of SHRI as best realized through a transdisciplinary process.

Yet, with the ideal disciplinary organization of SHRI still debated and underway, most scientific efforts in the field take on the form of multidisciplinary research (Baxter et al., 2016), and while many domains of research are surely contributing with a wide array of different theories and methods to SHRI research, examples of synthesis between these theories and methods across domains has only recently begun to emerge. This is not to say that these contributing research domains are entirely discrete; within SHRI, concepts such as anthropomorphism, empathy, trust, attachment, etc., are often found to have anchoring within a number of contributing disciplines or domains—just as the concept of novelty. Thus, from within the current state of multidisciplinarity, it could appear as though these concepts are functioning as points of transactional convergence between the contributing areas. One way to analyze the informational value of such points of convergence, is through the concept of “Boundary Objects” (BOs). A BO refers to those objects (concrete or abstract) that function as transactional instruments or touching-points between the different domains of practice of various stakeholders or contributors in a collaboration (be it organizational, entrepreneurial, scientific, etc.) (Leigh Star, 2010). Originally described^12^ by Star and Griesemer (1989) thus:

“This is an analytic concept of those scientific objects which both inhabit several intersecting social worlds […] and satisfy the informational requirements of each of them. Boundary objects are objects which are both plastic enough to adapt to local needs and the constraints of the several parties employing them. They are weakly structured in common use, and become strongly structured in individual-site use. These objects may be abstract or concrete. They have different meanings in different social worlds but their structure is common enough to more than one world to make them recognizable, a means of translation” (Star and Griesemer 1989: 393).

Since its introduction, the concept has been popular as an analytical tool for the dynamics of collaboration across borders of practice and knowledge, as well as much debated due to the ambiguity with which it was originally defined (Lee, 2007; Fujimura, 2010; Leigh Star, 2010). However, popularly construed, the power of the BO lies in its facilitation of collaboration between different stakeholders without consensus (Leigh Star, 2010). This facilitation is thought to arise through the object’s placement in the collaboration- or problem-space in a way that provides a relatively coarse-grained common understanding of the object within the collaborative field, while also allowing each contributor to maintain their finer-grained, domain-specific understandings without it hindering the overall objective. In this way, the lack of consensus concerns the fact that domain-specific notions and interpretations are allowed to differ (Fujimura, 2010; Leigh Star, 2010). Through the lens of this description, many of the concepts currently employed within SHRI could be viewed as BOs^13^, acting as convergent points around which smaller subareas of SHRI research form and exchange efforts.

However, if we are to not only bring together efforts of novelty research, but also foster the development of a larger, shared framework of novelty to integratively guide these efforts, the non-consensual premise of BOs will not do.

As exemplified in Section 2, research on novelty throughout the larger scientific landscape employs a multitude of differing definitions, descriptions, synonyms, methodologies, investigatory foci, and levels of analysis from which to approach the phenomenon of psychological novelty. While there are certainly larger trajectories of commonality between these approaches indicating an underlying phenomenon, there are, as illustrated, also conditions of unclarity and divergence both within and between areas. As such, these threads of novelty research do not effortlessly combine to reveal a systematic or coherent insight into the foundations and complexities of psychological novelty. It seems plausible to assume that the scattered and decentralized distribution of novelty research in general is reproduced in the current multidisciplinary organization of SHRI; while the phenomenon of robot sociality will probably require interdisciplinary, even transdisciplinary, efforts of scientific integration to be properly understood, we are only at the beginning of establishing the frames for such efforts. At this stage, the functioning of shared concepts and theories as BOs seems unproblematic and presumably even fruitful in facilitating initial contact between domains. Yet, the coarse granulation of the shared dimensions of BOs may result in the unquestioned retainment and reproduction of domain-specific idiosyncrasies and assumptions (Fujimura, 2010; Baggio et al., 2015). As the field of SHRI hopefully increases interdisciplinary efforts, operating in the shared space with a coarse, non-consensual^14^ understanding of novelty may lead to the presumed “adequacy” of internal novelty-concepts going largely unchallenged, thus failing to reveal both a need for a richer, shared concept as well as potential inconsistencies between the different, existing domain-specific concepts^15^.

Therefore, working towards developing a comprehensive, consensual, and interdisciplinary framework of novelty may be a substantial step along the transformational path to the realization of SHRI as an interdiscipline, if not transdiscipline.

### 4.3 Third Reason: An Integrative Framework for Novelty Research May Open Up New Interpretations of Results in SHRI

With a more nuanced and rich conception of novelty phenomena as central to the way in which human beings cope with the ambiguous experience of robot sociality, a new dimension of interpretation unfolds. This dimension may help to shed new light on ruling assumptions within SHRI as well as in generating alternative explanations for particular observations and pointing to new ways of approaching them.

In Smedegaard (2019), I offered examples of this utility by noting, in summary:

1) That understanding the central phenomenon of anthropomorphization in human-robot interactions in light of human responses to uncertainty and ambiguity implies that some levels and forms of anthropomorphizing may not be given nor stable, but instead change as social robots become less disruptive to sense-making.
2) That novelty as challenges in sense-making offers an additional view on how the mode of engagement affects the evaluation of and meaning ascribed to the social robot, as different modes of engagement provide different affordances for perception, action, and interaction, that all influence how the human experiences and acts upon the uncertainty and ambiguity of the situation.
3) That the acknowledgement of the centrality of novelty effects challenges the popular assumption about a causal relation between observations of how people seem to treat social robots and conclusions of how social robots should be designed, as the circumstances of novelty effects draws out the contingency and mouldability of current responses to social robots.

Another example not presented previously is found in the growing body of research on social robots and human creativity, because while it has been hypothesized that social robots may facilitate human creativity, this proposal does not seem to have been coupled particularly with robot novelty: Kahn et al. (2016) find support for the hypothesis, proposing that the robot effect on creativity was based on either the social presence of the robot itself, the additional language cues provided by the robot (as opposed to the PowerPoint-condition), or by its introduction and generation of an “Interaction Pattern Design”, previously proposed to support creativity (Kahn et al., 2016). In another study, Alves-Oliveira et al. (2019) failed to find support for the hypothesis, but nonetheless proposed in equal fashion [and with reference to Lubart’s proposals on computers as facilitators of creativity (Alves-Oliveira et al., 2019; Lubart, 2005)] that a potential robot-effect would arise on basis of the robot’s ability to encourage and support creativity through suggestion, feedback, and maintenance of attention. What is interesting in these proposals is the (implicit) placement of novelty primarily on the output-side of the creative process. If the field of SHRI was to operate with more prominent and nuanced attention to novelty effects, then perhaps these proposals would equally contain an identification of robot novelty as an instigator of these outputs. In a study by Druckman et al. (2021), the authors reflect upon the possibility of positive robot effects on creative negotiation outcomes as brought on by robot novelty effects on participant abilities to engage in divergent or creative thinking. Considering the arguments presented in this paper, that novelty may disrupt established meaning-making and force the experiencee in generating alternative meaning, such a reflection seems justified.

Framing robot facilitation of human creativity as (at least in part) a novelty effect, offers new investigatory and interpretational dimensions: for instance, placing the role of novelty as not only part of the produced output, but also as part of the generation itself brings forth an additional dimension from which to investigate novelty effects as cognitive processes, to which research on Innovation and creativity may offer useful measurements and theories. In addition, focus on novelty as a product rather than a “response-instigator,” may have the unfortunate consequence that robot effects on creativity are treated as universal or as a property of the robot. As such, a shared framework that emphasizes novelty as an experiential property points to ways of investigating the circumstances that influence whether a given human-robot interaction may foster creativity or not: from the descriptions in Section 2, we saw that at least individual thresholds of uncertainty as well as personality dispositions seem to affect the emotional appraisal of novelty. While Alves-Oliveira et al. (2019) did measure conventional vs. unconventional thinking and found no significance (however, not from awareness of novelty), the fact that participants did not get a chance to establish an interactional “rhythm” or “bond” prior to the collaborative task [the way participants in Kahn et al.’s study did (Kahn et al., 2016)] could be suggested to have affected the appraisal of robot novelty negatively, thus suppressing novelty facilitation of creative thinking.

### 4.4 Fourth Reason: Laying the Groundwork for a More General, Transdisciplinary Approach to Novelty Research

Returning to the description in Section 3 of SHRI research as potentially best realized within a transdisciplinary format, the working out of a shared, comprehensive and conceptually rich framework of psychological novelty may be a future product of such a format, in that it will alter current circumstances of research not only within SHRI but also within the contributing, original research domains. As noted in Section 2, there currently seems to be no framework of this scope and integration proposed, and research on novelty and novelty effects remain largely specialized and decentralized throughout psychology-reliant domains of research in general. In addition, the concept of novelty seems to be amorphous in that relations between novelty and central phenomena such as surprise, familiarity, repetition, habituation, etc., remain both empirically and conceptually unclear across domains. As the novelty of social robots is proposed as radical and profound to a degree not often encountered in human history, research on novelty may, through social robots, have a unique opportunity to investigate the phenomenon more distinctly and for a longer period than usually possible. Therefore, the development of a meta- or transdisciplinary framework of psychological novelty may be both extremely opportune at the present time as well as significant for not only for SHRI research, but for novelty research in general.

## 5 Conclusion

Throughout the paper, I have sought to make the case that the currently scattered and narrow efforts of novelty research within SHRI can be analyzed as a mirroring of larger circumstances of heterogeneity and decentralization within novelty research across psychology-reliant domains of research in general. I have further argued that the novelty of social robots as radical disruptions of sense-making cannot be properly understood nor investigated if these circumstances are allowed to continue. Understanding social robots as disrupted sense-making implies that the subject-material and research target of SHRI is (currently) significantly tied to novelty on a particularly complex level as it provokes participants and researchers alike to re-think core assumptions about the world in a way that may impact fundamental structures of social reality and interpersonal relations in the future. In light of this, we need to recognize the interdisciplinary efforts needed in order to comprehend and investigate the phenomenon within the scope and scale proportionate to its radicality. The field of SHRI needs to develop, through joint collaboration and integration, a conceptual framework of psychological novelty from which it is possible to identify, investigate, interpret and utilize the processes of novelty currently at play in social human-robot interactions. Such a framework could both yield greater comprehension of novelty occurrences and responses, as well as facilitate the centralization, integration, and utilization of novelty-knowledge across the scientific landscape, furthering more nuanced, comparable and generative approaches to novelty within SHRI studies.

One might object that social robot development is not nearly as far as my definition of them in Section 3 would suggest. Perhaps we do not need to be too concerned with these novelty effects as radical, because when these social agents are finally realized on a larger scale, the development and implementation will have progressed just gradually enough that people will have gotten used to them little by little through incremental design improvements, media coverage, and scattered encounters. While this may be so, I still believe that we would be missing valuable information about the current establishment of foundations for later implementations, practices, and narratives, if we do not begin to investigate systematically and comprehensively how people and researchers alike already engage with (even just the thought of) social robots presently. The empirical observations of broken core dichotomies of being, that I used to argue the radical novelty of social robots, are not prophecies of the future but very real and presently unfolding phenomena documented in SHRI research. As such, it matters not whether future developments of social robotics will be able to realize the potential we see today; to the experience of novelty, it matters only that we are already responding to this potential. While we essentially cannot know how social robots will be implemented and employed in the future, operating with a framework of novelty on how something experientially goes from novel to familiar, from uncertain to certain, from unpredictable to predictable, from rare to normal, should provide us with insights and tools in the present to engage with the establishment of this future.

## Data Availability

The original contributions presented in the study are included in the article/Supplementary Material, further inquiries can be directed to the corresponding author.

## Footnotes

1 The article aims, inter alia, to demonstrate that the concepts of “novelty” and “novelty effects” are not unanimously defined in scientific discourse. In Section 2, I provide the reader with a selection of multiple scientific definitions of the concepts, and in Section 3, I offer a specific definition of psychological novelty as “disrupted sense-making” argued to be particularly useful within SHRI research. Due to this progression of “unpacking” the concepts throughout the paper, I refrain from the common practice of introductory definitions, in order to avoid priming effects and maintain the open question of what we actually mean by “novelty”.

2 In sum, any kind of novelty-predication the truth-value of which is independent of subjective appraisal.

3 That novelty involves an awareness of unfamiliarity seems to limit the phenomenon to conscious phases of cognitive processing. I grant the possibility that, depending on theoretical approach, there may be instances where novelty is detected and processed without ever reaching consciousness. Yet, the existence of such instances seems far removed from our intuitive understanding of novelty as something that disrupts and captures attention.

4 To add some further disclaimers: for the sake of perspicuity, I omit nuances, sometimes lumping positions together that one might have good reasons to distinguish in other contexts, and other times separating positions that may in other contexts be commonly taken together.

5 The term “psychology-reliant” is meant to denote all research domains touching upon or involving psychological phenomena, thus not limited to branches within classical psychology.

6 Meant as the subject or phenomenon that is the primary target of scientific observation.

7 The New Ontological Category (NOC) Hypothesis proposed by Kahn et al. (2011) and Kahn and Shen (2017) concurs with this claim but has somewhat different aim: Kahn and colleagues seek to demonstrate the plausibility that a new ontological category is emerging with the advent of increasingly “sophisticated social technological entities” such as social robots–that is, they focus on the emergence of a new kind of being in the world that 1) is novel not on basis of being experienced as such, but on basis of never having existed before, and 2) is thought be a permanent (as opposed to perhaps only temporary), future addition to the categories of being in the world. In contrast, my argument is less metaphysically inclined, as I “only” seek to argue how social robots may for now*,* but not necessarily for good, be experienced, but not necessarily ontologically concluded, to be novel. The difference is visible in the fact that Kahn and colleagues do not seem to make the connection between the apparently new way of categorizing being and the presence of potential novelty effects, as they readily propose observed participant responses as indicators of a new, permanent way of reasoning about the world.

8 A general distinction can be drawn between taxonomical organization, where these “core” categories are understood as being the superordinate categories within which everything else can be placed (Cisek and Kalaska, 2010), and action-based organization as presented in Barsalou (2003), where “core” categories are understood as being those representations that are most frequently validated and solidified.

9 Such findings counter the general claim of the “Computer are Social Actors” (CASA) paradigm, originally developed within Human-Computer Interaction (HCI) research in the 90s but since extended to robots, that people mindlessly apply the same social scripts, categories, and attributes in interactions with computers and social robots, as they do in interactions with other human beings (Nass and Moon, 2000; Gambino et al., 2020). If this claim were also true with regard to human interactions with social robots, then the present claim of experienced robot novelty would be false. The clincher here is the experiential dimension of the human side of the interaction: insofar as an individual does not experience any dissonance or uncertainty when encountering a social robot, and really does approach and interact with it without conscious reflections on what they are doing (here, I am placing the burden of evidence not on behavioural output but on the cognitive processes underlying them), then, by the logic of the three assumptions presented in Section 2, we should conclude that there is no psychological novelty at play in the given interaction—of the kind that practically matters, at least. However, the empirical data of human-robot interaction go against this CASA claim. The research literature referenced above attests not to mindlessness but to conscious reflection, periods of disorientation and explorative, tentative applications of “scripts”. The struggle to come to terms with the experience of entities straddling the living/non-living dichotomy is documented in literature on the Uncanny Valley [where experiences of uncertainty and dissonance are found both within as well as outside of the “dip” (e.g., Gray and Wegner, 2012; Rosenthal-von der Pütten and Krämer, 2015; Gahrn-Andersen, 2020)], as well as in research on long-term interaction changes in perceptions, expectations and usage over time (e.g., Koay et al., 2007; De Graaf et al., 2017; Stubbs et al., 2005). In fact, motivated by the realization that the CASA paradigm does not explain why people adjust their responses to technology over time, Gambino et al., 2020 recently tried to modify the CASA paradigm so that it also would cover these cases. Ironically, the attempt effectively results in countering the original thesis by admitting that people do, after all, seem to respond differently to social robots than to computers as well as other human beings. Thus, it would appear that the CASA paradigm does not offer a suitable interpretation of the data. However, the question deserves a much more nuanced discussion than can be contained in this paper, especially since the CASA claim has within the last 20 years come to denote a multitude of sub-claims, interpretations, and expansions that all need to be considered when delineating and assessing how concepts such as “mindlessness,” “sociality,” and “computers,” “robots,” “machines” and the like, have been defined and operationalized in studies from this perspective.

11 Understood here as “most frequently validated” in line with Barsalou (2003)’s description presented in a previous footnote.

12 Although Star did present the notion in 1988 as well (Leigh Star, 2010).

13 As abstract subjects of investigation, analogue to how Bowker and Star [as referenced by Lee (2007, p. 310)] describe a concrete bird-specimen as a BO between various collaborators in a museum setting.

14 In the sense of not striving towards achieving a finer-grained consensus.

15 This is not to say that heterogeneity and context-specificity should not be allowed. Neither that novelty should not be defined in broad strokes. The point is that unquestioned heterogeneity and unanchored coarse granulations forego the kind of interdisciplinary knowledge-integration that is needed to motivate and facilitate the development of a broader, consensual, and foundational frame from which to analyze and map these complexities of novelty.
